# Spiro­[1,3-dioxolane-2,3′-indolin]-2′-one

**DOI:** 10.1107/S1600536810016132

**Published:** 2010-05-12

**Authors:** Yan Meng, Yanqing Miao

**Affiliations:** a School of Environmental Engineering, Chang’an University, South Second Cycle Road 368#, Xi’an 710054, Shannxi, People’s Republic of China; b Department of Pharmacy, Xi’an Medical University, Hanguang Round No. 137, Xi’an 710021, Xi’an, People’s Republic of China

## Abstract

The title compound, C_10_H_9_NO_3_, was synthesized by the condensation reaction of isatin (systematic name 1*H*-indole-2,3-dione) with glycol in presence of *p*-toluene­sulfonic acid. The indol-2-one ring system is essentially planar [N—C—C—C torsion angle = 3.1 (2)°], and the 1,3-dioxolane ring is slightly distorted. The crystal structure exhibits inter­molecular N—H⋯O hydrogen bonds.

## Related literature

For the synthesis of the title compound, see: Santos *et al.* (2008 ▶). For the bioactivity of the title compound, see: Demosthenes *et al.* (1998 ▶); Rajopadhye & Popp (1988 ▶).
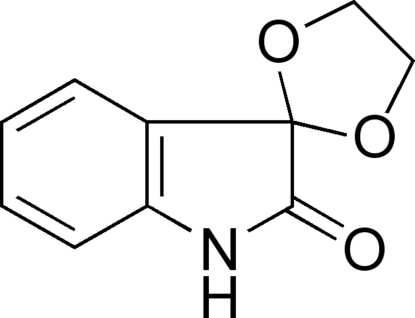


## Experimental

### 

#### Crystal data

C_10_H_9_NO_3_
*M* *_r_* = 191.18
Monoclinic, 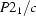

*a* = 7.484 (2) Å
*b* = 5.650 (1) Å
*c* = 20.942 (5) Å
β = 97.889 (8)°
*V* = 877.1 (4) Å^3^
*Z* = 4
Mo *K*α radiation
μ = 0.11 mm^−1^
*T* = 273 K
0.36 × 0.27 × 0.21 mm

#### Data collection

Bruker SMART CCD diffractometer
Absorption correction: multi-scan (*SADABS*; Bruker, 2002 ▶) *T*_min_ = 0.963, *T*_max_ = 0.989
4056 measured reflections
1534 independent reflections
1093 reflections with *I* > 2σ(*I*)
*R*_int_ = 0.070

#### Refinement

*R*[*F* ^2^ > 2σ(*F* ^2^)] = 0.048
*wR*(*F* ^2^) = 0.137
*S* = 1.09
1534 reflections
131 parameters
H atoms treated by a mixture of independent and constrained refinement
Δρ_max_ = 0.23 e Å^−3^
Δρ_min_ = −0.20 e Å^−3^

### 

Data collection: *SMART* (Bruker, 2002 ▶); cell refinement: *SAINT-Plus* (Bruker, 2002 ▶); data reduction: *SAINT-Plus*; program(s) used to solve structure: *SHELXS97* (Sheldrick, 2008 ▶); program(s) used to refine structure: *SHELXL97* (Sheldrick, 2008 ▶); molecular graphics: *SHELXTL* (Sheldrick, 2008 ▶); software used to prepare material for publication: *SHELXTL*.

## Supplementary Material

Crystal structure: contains datablocks I, global. DOI: 10.1107/S1600536810016132/lx2143sup1.cif

Structure factors: contains datablocks I. DOI: 10.1107/S1600536810016132/lx2143Isup2.hkl

Additional supplementary materials:  crystallographic information; 3D view; checkCIF report

## Figures and Tables

**Table 1:** Hydrogen-bond geometry (Å, °)

*D*—H⋯*A*	*D*—H	H⋯*A*	*D*⋯*A*	*D*—H⋯*A*
N1—H1⋯O1^i^	0.87 (3)	2.07 (3)	2.941 (3)	174 (2)

Symmetry code: (i) 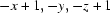.

**Fig. 1.**
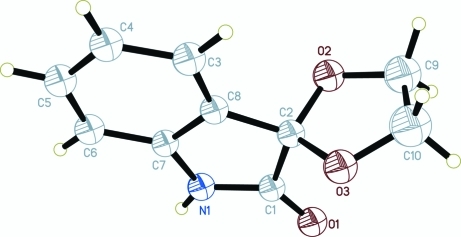
The molecular structure of the title compound with the atom numbering scheme. Displacement ellipsoids are drawn at the 30% probability level. H atoms are presented as a small cycles of arbitrary radius.

**Fig. 2.**
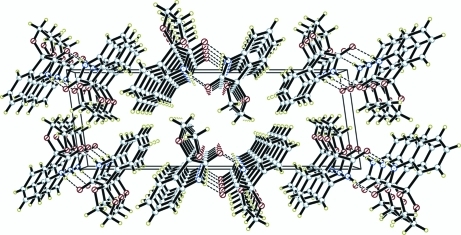
N–H···O interactions (dotted lines) in the crystal structure of the title compound.

## supplementary crystallographic information

### Comment

Isatin derivatives, has caught great attention of many researchers as a versatile lead molecule for designing of potential drugs for the variety of biological activities, such as anti-bacteria, anti-virus, anti-tumor and neuroprotection. Among these compounds, spiro-oxindol analogues have received considerable attention as potential anti-bacteria and neuroprotection agents(Demosthenes *et al.* 1998; Rajopadhye *et al.* 1988; Santos *et al.* 2008).

The X-ray structural analysis confirmed the assignment of its structure from spectroscopic data. The molecular structure is depicted in Fig. 1, and a diagram of interactions between the title compounds is depicted in Fig. 2. Geometric parameters of the title compound are in the usual ranges. The crystal packing (Fig. 2) is stabilized by intermolecular N—H···O hydrogen bonds between the indoline H atom and the oxygen of the C═O unit, with a N1—H1···O1^i^ (Table 1).

### Experimental

Isatin (1 mmol) and glycol (1 mmol) was dissolved in cyclohexane (20 ml), and 0.01 mmol TsOH was added. The mixture was stirred under reflux. After completion of the reaction, it was evaporated to dryness, followed by chromatography to the pure title compound. ^1^H-NMR (D_6_-Acetone, 400 MHz) delta: 10.44 (1*H*, s), 7.33 (2*H*, m), 7.00 (1*H*, td, *J* = 7.2, 0.8 Hz), 6.82 (1*H*, d, *J* = 7.6 Hz), 4.33 (2*H*, m), 4.23 (2*H*, m); EI–MS, m/z (%): 233 (M^+^)

### Refinement

The H atom bound N atom was located from difference Fourier map and refined freely. All H atoms of C atoms were positioned geometrically and refined using a riding model, with C–H = 0.93 Å for aryl and 0.97 Å for methylene H atoms. *U*_iso_(H)= 1.2*U*_eq_(C) for all H atoms.

### Crystal data

**Table:**

C_10_H_9_NO_3_	*F*(000) = 400
*M**_r_* = 191.18	*D*_x_ = 1.448 Mg m^−^^3^
Monoclinic, *P*2_1_/*c*	Mo *K*α radiation, λ = 0.71073 Å
Hall symbol: -P 2ybc	Cell parameters from 7304 reflections
*a* = 7.484 (2) Å	θ = 1.5–25.0°
*b* = 5.650 (1) Å	µ = 0.11 mm^−^^1^
*c* = 20.942 (5) Å	*T* = 273 K
β = 97.889 (8)°	Block, colourless
*V* = 877.1 (4) Å^3^	0.36 × 0.27 × 0.21 mm
*Z* = 4	

### Data collection

**Table:**

Bruker SMART CCD diffractometer	1534 independent reflections
Radiation source: fine-focus sealed tube	1093 reflections with *I* > 2σ(*I*)
graphite	*R*_int_ = 0.070
phi and ω scans	θ_max_ = 25.0°, θ_min_ = 2.0°
Absorption correction: multi-scan (*SADABS*; Bruker, 2002)	*h* = −8→8
*T*_min_ = 0.963, *T*_max_ = 0.989	*k* = −6→6
4056 measured reflections	*l* = −21→24

### Refinement

**Table:**

Refinement on *F*^2^	Primary atom site location: structure-invariant direct methods
Least-squares matrix: full	Secondary atom site location: difference Fourier map
*R*[*F*^2^ > 2σ(*F*^2^)] = 0.048	Hydrogen site location: difference Fourier map
*wR*(*F*^2^) = 0.137	H atoms treated by a mixture of independent and constrained refinement
*S* = 1.09	*w* = 1/[σ^2^(*F*_o_^2^) + (0.0648*P*)^2^ + 0.151*P*] where *P* = (*F*_o_^2^ + 2*F*_c_^2^)/3
1534 reflections	(Δ/σ)_max_ < 0.001
131 parameters	Δρ_max_ = 0.23 e Å^−^^3^
0 restraints	Δρ_min_ = −0.20 e Å^−^^3^

### Special details

**Table:**

Geometry. All esds (except the esd in the dihedral angle between two l.s. planes) are estimated using the full covariance matrix. The cell esds are taken into account individually in the estimation of esds in distances, angles and torsion angles; correlations between esds in cell parameters are only used when they are defined by crystal symmetry. An approximate (isotropic) treatment of cell esds is used for estimating esds involving l.s. planes.
Refinement. Refinement of F^2^ against ALL reflections. The weighted R-factor wR and goodness of fit S are based on F^2^, conventional R-factors R are based on F, with F set to zero for negative F^2^. The threshold expression of F^2^ > 2sigma(F^2^) is used only for calculating R-factors(gt) etc. and is not relevant to the choice of reflections for refinement. R-factors based on F^2^ are statistically about twice as large as those based on F, and R- factors based on ALL data will be even larger.

### Fractional atomic coordinates and isotropic or equivalent isotropic displacement parameters (Å^2^)

**Table:**

	*x*	*y*	*z*	*U*_iso_*/*U*_eq_	
O1	0.6983 (2)	0.2241 (3)	0.50491 (8)	0.0600 (5)	
O3	0.6788 (2)	0.6884 (3)	0.43906 (8)	0.0551 (5)	
O2	0.8104 (2)	0.4151 (3)	0.38133 (8)	0.0560 (5)	
N1	0.4575 (3)	0.1613 (4)	0.42609 (9)	0.0469 (6)	
H1	0.407 (4)	0.044 (5)	0.4439 (13)	0.070 (9)*	
C7	0.3813 (3)	0.2722 (4)	0.36864 (10)	0.0400 (6)	
C2	0.6494 (3)	0.4723 (4)	0.40675 (10)	0.0417 (6)	
C8	0.4869 (3)	0.4639 (4)	0.35588 (10)	0.0402 (6)	
C3	0.4343 (3)	0.6053 (4)	0.30311 (11)	0.0506 (6)	
H3A	0.5032	0.7355	0.2945	0.061*	
C6	0.2254 (3)	0.2139 (4)	0.32859 (11)	0.0512 (6)	
H6A	0.1566	0.0834	0.3370	0.061*	
C1	0.6076 (3)	0.2731 (4)	0.45352 (10)	0.0447 (6)	
C5	0.1752 (3)	0.3571 (5)	0.27544 (11)	0.0550 (7)	
H5A	0.0707	0.3216	0.2477	0.066*	
C4	0.2758 (3)	0.5498 (5)	0.26285 (11)	0.0542 (7)	
H4A	0.2381	0.6443	0.2272	0.065*	
C10	0.8530 (4)	0.7641 (6)	0.4348 (2)	0.0911 (11)	
H10A	0.9164	0.7972	0.4774	0.109*	
H10B	0.8504	0.9075	0.4092	0.109*	
C9	0.9435 (4)	0.5760 (6)	0.40444 (16)	0.0809 (10)	
H9B	1.0019	0.6383	0.3694	0.097*	
H9C	1.0340	0.5011	0.4355	0.097*	

### Atomic displacement parameters (Å^2^)

**Table:**

	*U*^11^	*U*^22^	*U*^33^	*U*^12^	*U*^13^	*U*^23^
O1	0.0568 (12)	0.0592 (12)	0.0595 (10)	−0.0091 (9)	−0.0079 (9)	0.0079 (8)
O3	0.0577 (11)	0.0369 (9)	0.0735 (11)	−0.0087 (8)	0.0196 (9)	−0.0187 (8)
O2	0.0441 (10)	0.0489 (10)	0.0777 (11)	−0.0054 (8)	0.0176 (8)	−0.0210 (8)
N1	0.0488 (13)	0.0362 (11)	0.0535 (11)	−0.0084 (10)	−0.0008 (9)	0.0106 (9)
C7	0.0435 (14)	0.0317 (12)	0.0452 (12)	0.0029 (10)	0.0081 (10)	0.0008 (9)
C2	0.0424 (13)	0.0305 (12)	0.0538 (12)	−0.0037 (10)	0.0127 (10)	−0.0069 (10)
C8	0.0435 (13)	0.0297 (12)	0.0490 (12)	0.0015 (10)	0.0122 (10)	−0.0006 (9)
C3	0.0542 (15)	0.0397 (13)	0.0605 (14)	0.0000 (11)	0.0174 (12)	0.0095 (11)
C6	0.0495 (16)	0.0433 (14)	0.0596 (14)	−0.0058 (11)	0.0032 (11)	0.0019 (11)
C1	0.0464 (14)	0.0368 (13)	0.0497 (12)	0.0005 (11)	0.0022 (11)	−0.0019 (10)
C5	0.0483 (15)	0.0612 (17)	0.0540 (13)	0.0034 (13)	0.0014 (11)	0.0042 (12)
C4	0.0558 (16)	0.0569 (16)	0.0505 (13)	0.0139 (14)	0.0095 (11)	0.0114 (11)
C10	0.059 (2)	0.0579 (19)	0.160 (3)	−0.0178 (16)	0.028 (2)	−0.043 (2)
C9	0.0602 (18)	0.084 (2)	0.102 (2)	−0.0233 (17)	0.0217 (16)	−0.0377 (19)

### Geometric parameters (Å, °)

**Table:**

O1—C1	1.223 (2)	C3—C4	1.393 (3)
O3—C10	1.387 (4)	C3—H3A	0.9300
O3—C2	1.399 (3)	C6—C5	1.386 (3)
O2—C9	1.386 (3)	C6—H6A	0.9300
O2—C2	1.420 (3)	C5—C4	1.370 (3)
N1—C1	1.347 (3)	C5—H5A	0.9300
N1—C7	1.406 (3)	C4—H4A	0.9300
N1—H1	0.87 (3)	C10—C9	1.452 (4)
C7—C6	1.380 (3)	C10—H10A	0.9700
C7—C8	1.388 (3)	C10—H10B	0.9700
C2—C8	1.503 (3)	C9—H9B	0.9700
C2—C1	1.552 (3)	C9—H9C	0.9700
C8—C3	1.377 (3)		
			
C10—O3—C2	108.95 (19)	C5—C6—H6A	121.2
C9—O2—C2	109.03 (19)	O1—C1—N1	126.8 (2)
C1—N1—C7	111.8 (2)	O1—C1—C2	125.8 (2)
C1—N1—H1	124.0 (17)	N1—C1—C2	107.44 (18)
C7—N1—H1	123.8 (17)	C4—C5—C6	121.5 (2)
C6—C7—C8	121.7 (2)	C4—C5—H5A	119.2
C6—C7—N1	128.6 (2)	C6—C5—H5A	119.2
C8—C7—N1	109.75 (19)	C5—C4—C3	120.5 (2)
O3—C2—O2	107.13 (17)	C5—C4—H4A	119.8
O3—C2—C8	115.39 (18)	C3—C4—H4A	119.8
O2—C2—C8	111.88 (17)	O3—C10—C9	107.6 (2)
O3—C2—C1	111.09 (17)	O3—C10—H10A	110.2
O2—C2—C1	109.05 (18)	C9—C10—H10A	110.2
C8—C2—C1	102.17 (17)	O3—C10—H10B	110.2
C3—C8—C7	120.0 (2)	C9—C10—H10B	110.2
C3—C8—C2	131.7 (2)	H10A—C10—H10B	108.5
C7—C8—C2	108.34 (17)	O2—C9—C10	106.1 (2)
C8—C3—C4	118.7 (2)	O2—C9—H9B	110.5
C8—C3—H3A	120.6	C10—C9—H9B	110.5
C4—C3—H3A	120.6	O2—C9—H9C	110.5
C7—C6—C5	117.6 (2)	C10—C9—H9C	110.5
C7—C6—H6A	121.2	H9B—C9—H9C	108.7
			
C1—N1—C7—C6	−177.7 (2)	C7—C8—C3—C4	−0.9 (3)
C1—N1—C7—C8	1.5 (3)	C2—C8—C3—C4	178.4 (2)
C10—O3—C2—O2	−0.6 (3)	C8—C7—C6—C5	−1.3 (4)
C10—O3—C2—C8	−125.9 (3)	N1—C7—C6—C5	177.8 (2)
C10—O3—C2—C1	118.4 (3)	C7—N1—C1—O1	176.7 (2)
C9—O2—C2—O3	7.5 (3)	C7—N1—C1—C2	−5.3 (3)
C9—O2—C2—C8	134.9 (2)	O3—C2—C1—O1	−51.7 (3)
C9—O2—C2—C1	−112.8 (2)	O2—C2—C1—O1	66.2 (3)
C6—C7—C8—C3	1.8 (3)	C8—C2—C1—O1	−175.3 (2)
N1—C7—C8—C3	−177.5 (2)	O3—C2—C1—N1	130.3 (2)
C6—C7—C8—C2	−177.7 (2)	O2—C2—C1—N1	−111.9 (2)
N1—C7—C8—C2	3.1 (2)	C8—C2—C1—N1	6.7 (2)
O3—C2—C8—C3	54.2 (3)	C7—C6—C5—C4	−0.1 (4)
O2—C2—C8—C3	−68.6 (3)	C6—C5—C4—C3	0.9 (4)
C1—C2—C8—C3	174.9 (2)	C8—C3—C4—C5	−0.4 (4)
O3—C2—C8—C7	−126.4 (2)	C2—O3—C10—C9	−6.2 (4)
O2—C2—C8—C7	110.7 (2)	C2—O2—C9—C10	−11.1 (4)
C1—C2—C8—C7	−5.8 (2)	O3—C10—C9—O2	10.7 (4)

### Hydrogen-bond geometry (Å, °)

**Table:**

*D*—H···*A*	*D*—H	H···*A*	*D*···*A*	*D*—H···*A*
N1—H1···O1^i^	0.87 (3)	2.07 (3)	2.941 (3)	174 (2)

Symmetry codes: (i) −*x*+1, −*y*, −*z*+1.
